# Quantifying the Molecular Origins of Opposite Solvent Effects on Protein-Protein Interactions

**DOI:** 10.1371/journal.pcbi.1003072

**Published:** 2013-05-16

**Authors:** Vincent Vagenende, Alvin X. Han, Han B. Pek, Bernard L. W. Loo

**Affiliations:** Bioprocessing Technology Institute, A*STAR (Agency for Science, Technology and Research), Singapore; Max Planck Institute for Biophysical Chemistry, Germany

## Abstract

Although the nature of solvent-protein interactions is generally weak and non-specific, addition of cosolvents such as denaturants and osmolytes strengthens protein-protein interactions for some proteins, whereas it weakens protein-protein interactions for others. This is exemplified by the puzzling observation that addition of glycerol oppositely affects the association constants of two antibodies, D1.3 and D44.1, with lysozyme. To resolve this conundrum, we develop a methodology based on the thermodynamic principles of preferential interaction theory and the quantitative characterization of local protein solvation from molecular dynamics simulations. We find that changes of preferential solvent interactions at the protein-protein interface quantitatively account for the opposite effects of glycerol on the antibody-antigen association constants. Detailed characterization of local protein solvation in the free and associated protein states reveals how opposite solvent effects on protein-protein interactions depend on the extent of dewetting of the protein-protein contact region and on structural changes that alter cooperative solvent-protein interactions at the periphery of the protein-protein interface. These results demonstrate the direct relationship between macroscopic solvent effects on protein-protein interactions and atom-scale solvent-protein interactions, and establish a general methodology for predicting and understanding solvent effects on protein-protein interactions in diverse biological environments.

## Author Summary

Solvents play a fundamental role in living systems where they mediate the interactions between proteins and other biomolecules. Besides water, biological solvents often contain high concentrations of small molecular compounds known as cosolvents. Although many studies have reported specific and opposite effects of cosolvents on protein-protein interactions, the molecular origins of this phenomenon remain unknown. In this study, we develop a methodology to predict solvent effects on protein-protein interactions by computational characterization of local protein solvation. We use this methodology to explain the opposite effects of glycerol on the binding affinity of two antibodies. Quantitative characterization of local solvation near the protein-protein interface reveals that solvation changes not only depend on the extent of dewetting of the protein-protein contact region, but also on specific protein structural changes at the periphery of the protein-protein interface. Our results demonstrate the direct relationship between solvent effects on protein-protein interactions and local solvent-protein interactions, and establish a general methodology for predicting and understanding cosolvent effects on protein-protein interactions in diverse biological environments.

## Introduction

Cosolvents such as denaturants, salts, amino acids and polyols play an important role in many protein processes as they modify the strength of intra- and intermolecular interactions of proteins in various cellular and biochemical environments [1]–[5]. Cosolvents that strengthen protein-protein interactions induce macromolecular assembly and increase the conformational stability of proteins [4], [6]; cosolvents that weaken protein-protein interactions generally increase protein solubility and may prevent the formation of protein aggregates with undesired immunological or pathological properties [7], [8]. Despite the growing evidence for the importance of cosolvents in regulating biological processes [9]–[11] and the widespread use of cosolvents in protein formulation and refolding [1], [2], [12]–[16], general understanding of cosolvent effects on protein interactions is lacking and optimizing solvent conditions for a particular protein process typically requires laborious empirical screening of various cosolvents.

Preferentially excluded cosolvents generally stabilize proteins, whereas cosolvents that preferentially interact with the protein surface often destabilize and denature proteins [6], [17]. Similarly, it is often implied that preferentially excluded cosolvents increase protein-protein interactions, whereas cosolvents that preferentially interact with the protein surface weaken protein-protein interactions. This dichotomy is, however, irreconcilable with many studies in literature that report specific – and even opposite – effects of cosolvents on protein-protein interactions [9], [18]–[23]. For instance, osmolytes such as glycerol and TMAO increase fibril formation of Aβ-peptide involved in Alzheimer's disease, but decrease aggregation of ataxin-3 involved in Machado-Joseph disease [18]. Another study reports that glycerol promotes the association of cytochrome c with cytochrome b5 but inhibits the association of cytochrome c and cytochrome c oxidase [19]. Yet another study reports a more than tenfold decrease of antibody-antigen binding affinity measured in vivo compared to the corresponding value measured in vitro [24]. This example not only illustrates how protein-protein interactions differ in distinct solution environments, but also calls for caution in correlating pharmacological properties to protein-protein interactions data measured in vitro [25]. Taken together, these studies highlight that a general approach for understanding cosolvent effects on protein interactions should account for specific solvent-protein interactions.

Current understanding of cosolvent effects on protein interactions is largely derived from the principles of linked functions [26] and the thermodynamic theory of preferential interactions in multicomponent solutions [27]–[36]. These principles dictate that the addition of cosolvent will shift the association constant K_A_ of two proteins towards the protein state with the highest preferential interaction coefficient 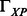 [6], [37], [38]:
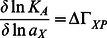
(1)In Eq. 1, 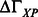 is the difference of the preferential interaction coefficients of the associated and free protein states, and a_x_ is the activity of the cosolvent. This equation directly relates cosolvent effects on the association constant with solvation changes upon association. Unfortunately, application of Eq. 1 for understanding cosolvent effects on protein processes has been incapacitated because of the difficulty to obtain precise values of preferential interaction coefficients 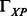 for distinct protein states [39], [40], and because 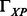, which quantifies preferential interactions averaged over the entire protein surface, does not provide information on local solvation properties at distinct loci of the protein surface [6], [40].

Here we develop and validate a methodology to quantify the molecular origins of opposite solvent effects on protein-protein interactions. By combining the thermodynamic principles of preferential interaction theory with surface plasmon resonance experiments and computational characterization of local protein solvation, we demonstrate the direct relationship between macroscopic solvent effects on protein-protein interactions and atom-scale solvent-protein interactions. We apply this methodology to understand the opposite effects of glycerol on the association constants of two antibodies - D1.3 and D44.1 - with lysozyme, and we find that cosolvent-effects on protein-protein interactions critically depend on the extent of dewetting of the protein-protein contact region and on local structural changes of the protein that alter cooperative solvent-protein interactions through multiple hydrogen-bonds.

## Results

### Opposite effects of glycerol on protein-protein interactions

**Figure 1:**
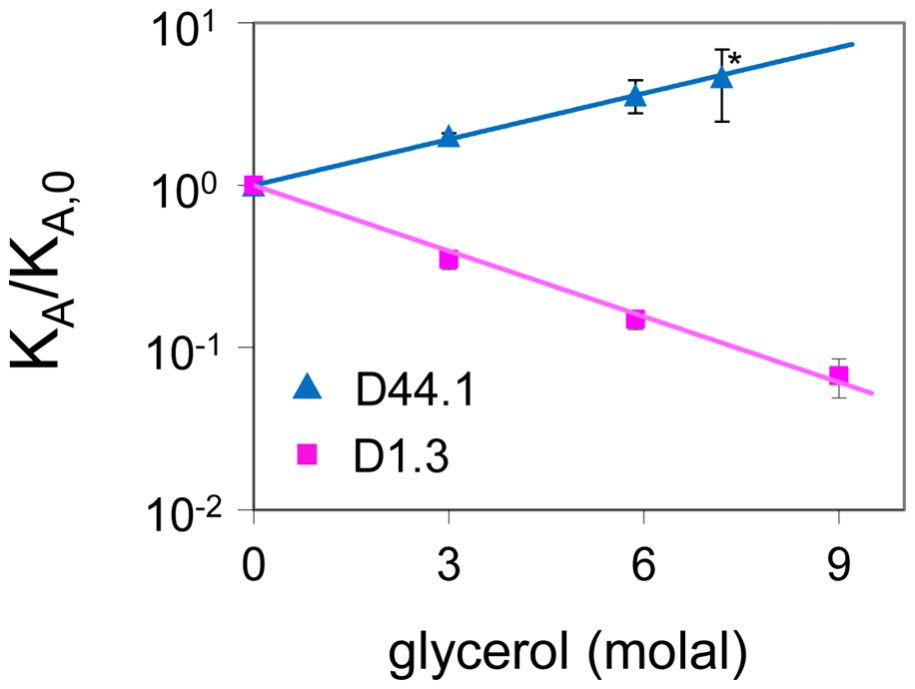
Opposite effects of glycerol on the association constant K_A_ of Fab D44.1 and scFv D1.3 with lysozyme. K_A_/K_A,0_ is the ratio of the association constants with and without glycerol. The data point marked with an asterisk is derived from Goldbaum et al. [21] and all other data points are determined by surface plasmon resonance.

To gain understanding in the molecular origins of opposite solvent effects on protein-protein interactions, we focus on a pertinent example of opposite effects of glycerol on the association constants of two different antibodies with lysozyme [21]. We use surface plasmon resonance to characterize the opposite effects of glycerol on the association constants of antibody fragments D1.3 and D44.1 over a wide concentration range (0–9 molal glycerol). Figure 1 shows that the association constant of the D1.3-lysozyme complex decreases exponentially with respect to glycerol molality, whereas the association constant K_A_ of the D44.1-lysozyme complex increases exponentially with glycerol molality. Exponential responses of equilibrium constants with respect to cosolvent concentrations have also been observed for other protein binding and unfolding reactions, and it has been suggested that the underlying mechanisms are closely related [17]. Such a common mechanism could stem from the thermodynamic principles of preferential interaction theory, yet evidence for this hypothesis is lacking.

To find out whether opposite solvent effects on protein-protein interactions can be understood from preferential interaction theory, we investigate whether Eq. 1 is able to explain the opposite effects of glycerol on the association constants of D1.3 and D44.1. Taking into account the exponential responses of the association constants with respect to glycerol molality (Figure 1), Eq. 1 can be simplified into the following equation (Text S1):
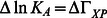
(2)This equation dictates that the change of the logarithms of the association constant K_A_ upon addition of glycerol equals the difference of preferential interaction coefficients 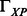 of the associated and free protein states. Application of Eq. 2 thus requires 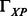-values of the associated and free states of D1.3, D44.1 and lysozyme in aqueous glycerol.

### Preferential solvent interactions of free and associated proteins

**Figure 2:**
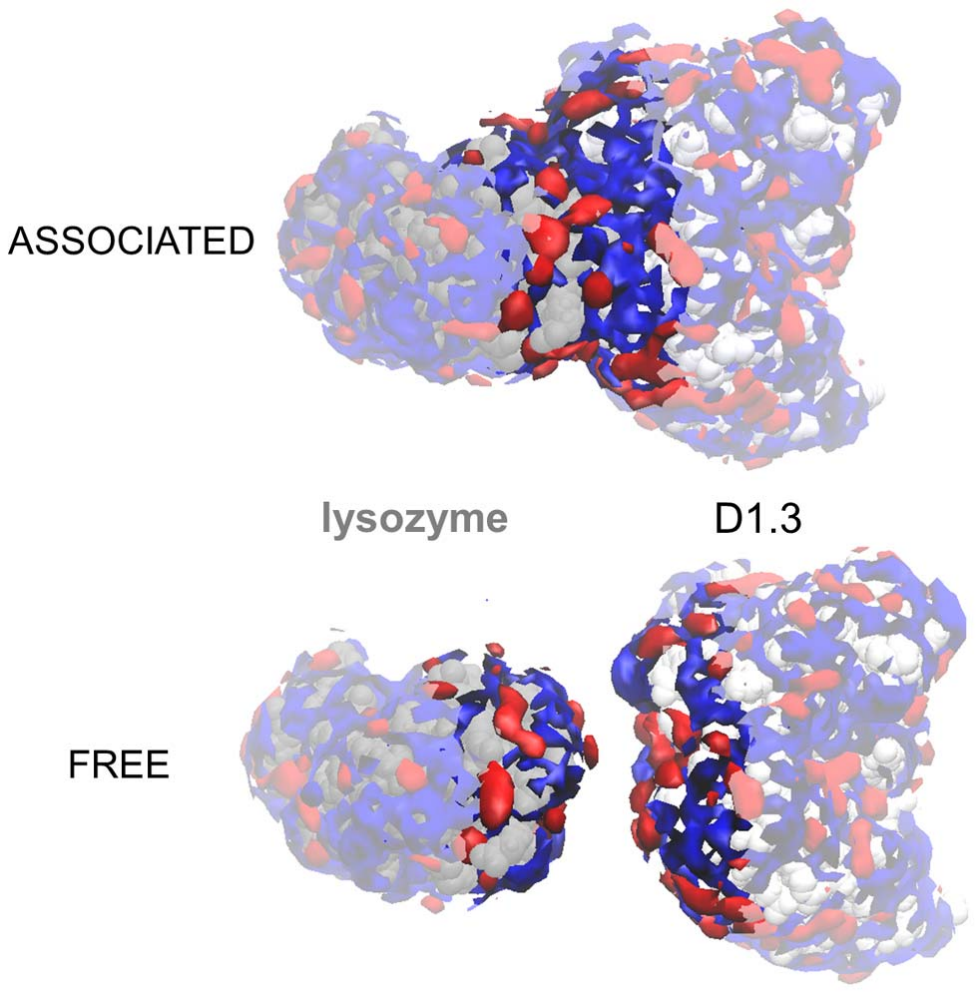
Local concentration maps of lysozyme and D1.3 in the associated and free states. Solvent regions that are preferentially solvated by glycerol and water are colored in red and blue respectively, and solvent regions near the interface region are highlighted.

To quantify 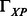-values of the free and associated protein states of D1.3, D44.1 and lysozyme in aqueous glycerol, we performed six independent molecular dynamics simulations for the respective protein systems. 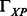-values of all proteins and protein-complexes are negative (Table 1), indicating overall exclusion of glycerol for all proteins. Differences of 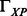-values between the associated and free protein states are relatively small and subject to large standard errors (Table 1). To improve the precision of computed 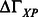-values, we identified protein surface regions where local solvation differs in the associated and free protein state. Local concentration maps of the free and associated protein states differ markedly near the protein-protein interface region, but not for the rest of the protein surface (Figure 2 and Figure S1). This indicates that protein-protein association only affects solvation near the protein-protein interface.

**Table 1:** Preferential interaction coefficients of free and associated proteins in 6 molal glycerol.

	D1.3	lysozyme	D1.3-lysozyme	D44.1	Lysozyme	D44.1-lysoyzme
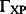	−11.1±0.4	−5.2±0.8	−20.6±1.5	−28.2±1.5	−6.9±0.8	−33.5±1.9
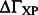 a		−4.3±1.7			1.7±2.5	
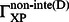 b	−9.2±0.4	−3.5±0.5	−13.9±1.3	−26.0±1.6	−4.7±0.7	−31.2±1.7
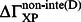 a		−1.2±1.5			−0.5±2.4	
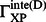 b	−1.9±0.2	−1.8±0.2	−6.7±0.5	−2.1±0.4	−2.2±0.4	−2.3±0.6
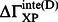 a		−3.0±0.6			2.0±0.9	
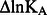 c		−2.3±0.1			1.2±0.3	

a Difference of preferential interaction coefficients between associated and free proteins.

b Regional preferential interaction coefficients at the protein-protein interface region inte(D) and the complementary protein surface region non-inte(D). The distance D is 7 Å for the D1.3-lysozyme complex and 9 Å for the D44.1-lysozyme complex.

c Calculated from experimental K_A_-values at 6 molal glycerol (Figure 1).

**Figure 3:**
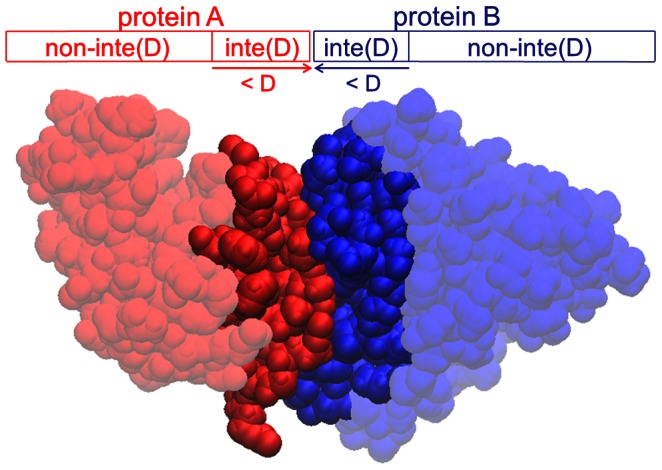
Definition of the interface region inte(D) of a protein-protein complex. The interface region inte(D) of protein A is defined as the continuous protein surface region comprising all residues with at least one atom within a distance D from protein B. All other residues of protein A belong to the complementary region, non-inte(D).

Since solvation changes upon protein-protein association are limited to protein surface regions near the protein-protein surface, the difference of 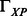-values between the associated and free protein states could be calculated from local preferential interaction coefficients near the protein-protein interface. We define the protein-protein interface region inte(D) as the contiguous protein surface region comprising all residues of the protein-protein complex with at least one atom within a distance D from the associated protein (Figure 3). All protein residues outside inte(D) are grouped into the complementary region non-inte(D), and the following equation is automatically met [40]:
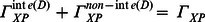
(3)In the above equation, 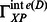 and 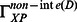 are the regional preferential interaction coefficients of the interface region inte(D) and the complementary surface region, respectively. The distance D is determined as the minimal distance at which values of 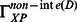 do not significantly differ between the free and associated proteins (Table 1), and we get 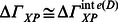. Notably, 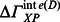-values have a higher precision than the corresponding 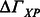-values (Table 1).

The association of D1.3 with lysozyme results in an overall decrease in preferential interaction coefficients (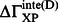
<0), whereas the association of D44.1 with lysozyme results in an overall increase in preferential interaction coefficients upon protein-protein association (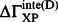
>0) (Table 1). Strikingly, the values of 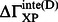 quantitatively agree with experimentally determined changes of the association constant, 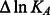 (Table 1). This agreement conforms with Eq. 2 and establishes the direct relationship between protein solvation and solvent effects on protein-protein interactions. Although the theoretical foundations of this relationship – i.e. the thermodynamic principles of linked function and preferential interactions theory - have been established over the past decades [26]–[36], empirical evidence supporting this relationship is lacking and the extent to which other solvent-related factors, such as the dielectric constant and viscosity of the solvent [41], (co-)determine cosolvent effects on protein-protein interactions remain unknown. Our finding that cosolvent effects on protein-protein association constants quantitatively agree with changes in preferential interaction coefficients between the associated and free protein states pinpoints the predominant role of preferential solvent interactions in determining the effects of cosolvents on protein-protein interactions.

Having established the direct relationship between solvent effects on protein-protein interactions and preferential solvent interactions at the protein-protein interface , we can now address the first part of the conundrum of opposite glycerol effects on the association constants of D1.3 and D44.1: glycerol weakens binding of D1.3 with lysozyme because of the overall decrease of preferential interaction coefficients upon antibody-antigen association, but glycerol strengthens binding of D44.1 with lysozyme because of the overall increase of preferential interaction coefficients upon antibody-antigen association (Table 1). This raises, however, another pertinent question: why does the association of D1.3 with lysozyme result in an overall decrease of preferential interactions with glycerol, whereas the association of D44.1 with lysozyme results in an overall increase of preferential interactions with glycerol? To address this question, we further analyze protein-association related changes of local solvation near the protein-protein interface of D1.3, D44.1 and lysozyme.

### Solvation at the protein-protein interface

**Figure 4:**
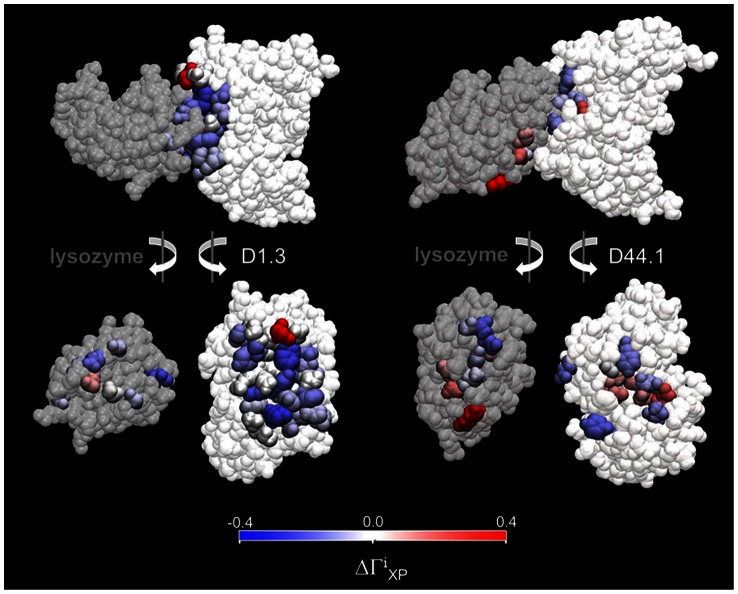
Local changes in preferential interactions upon protein-protein association of D1.3 and D44.1 with lysozyme. Residues for which the local preferential interaction coefficient 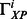 is greater (smaller) for the associated than for the free proteins are colored red (blue). For clarity, only the V_H_ and V_L_ regions of the antibody fragments are displayed.

**Figure 5:**
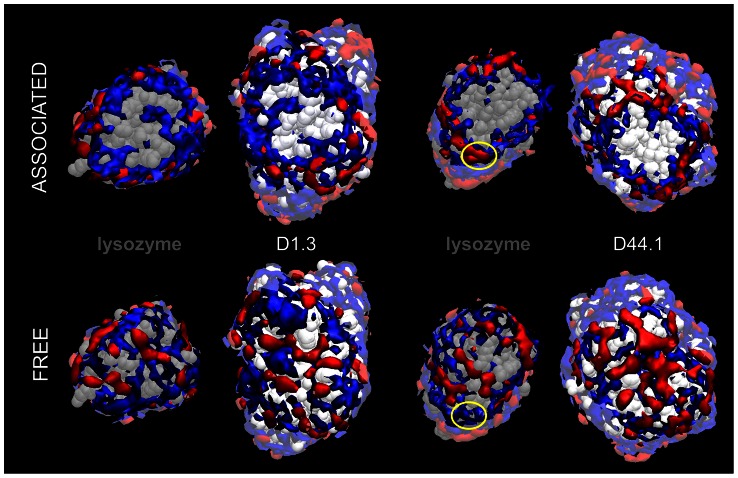
Solvation of the interface regions of D1.3, D44.1 and lysozyme in the associated and free states. Solvent regions that are preferentially solvated by glycerol (water) are colored in red (blue). The yellow circle indicates the protein surface locus near the N-terminus of lysozyme.

**Figure 6:**
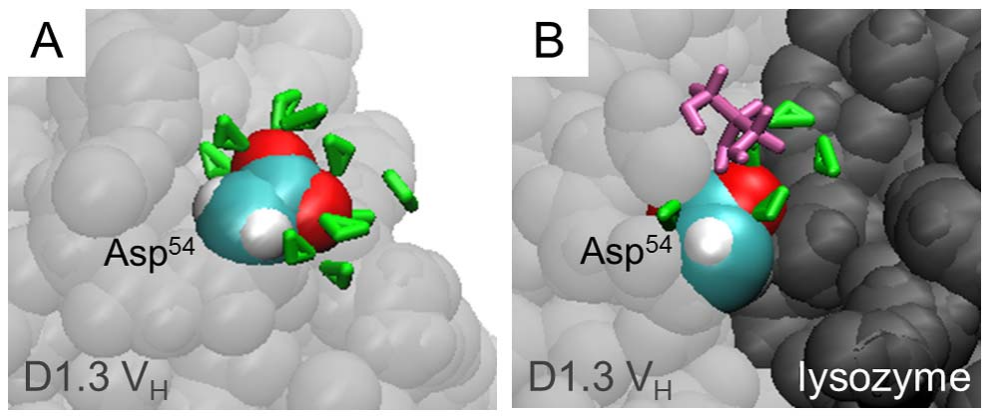
Snapshot of solvent molecules near Asp^54^ of D1.3 V_H_ in the free state (A) and associated to lysozyme (B). Water and glycerol molecules within 5 Å from Asp^54^ are represented in green and purple, respectively. C- and O-atoms of the side-chain of Asp54 are highlighted in cyan and red, respectively.

The global preferential interaction coefficient of a protein, 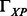, is the sum of the local preferential interaction coefficients 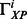 of all protein residues that comprise the protein surface [40], [42]. Changes of 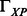 upon protein association can therefore be attributed to differences of 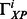 in the free and associated protein states. For the D1.3-lysozyme complex, protein-protein association leads to a decrease of 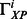 for all residues that are buried at the protein-protein contact region (Figure 4 and Figure S2). This is because, unlike water, glycerol is totally excluded from the protein-protein contact (Figure 5). Similarly, most residues at the periphery of the contact region of the D1.3-lysozyme complex see a decrease of 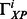-values in the associated state (colored in blue Figure 4). The only exception is Asp^54^ of the V_H_-chain of D1.3, which is strongly preferentially hydrated in the free state but only moderately preferentially hydrated as its side chain becomes partially buried in the associated state (Figure 6 and Figure S2). The positive contribution of Asp^54^ to 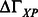 is, however, significantly smaller than the sum of the negative contributions of the other interface residues. As a result, 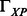 decreases upon association of D1.3 with lysozyme.

For the D44.1-lysozyme complex, changes of local preferential interactions upon protein-protein association are more balanced with values of 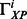 increasing for some residues and decreasing for others (Figure 4 and Figure S3). Similar to the D1.3-lysozyme complex, most residues with significant changes of 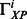 are found near the protein-protein contact region (Figure 4 and Figure S3). However, unlike the D1.3-lysozyme complex, the contact region of the D44.1-lysozyme complex is mostly dry (Figure 5). Changes of 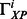 for residues at the contact region of the D44.1-lysozyme complex thus reflect the loss of preferential solvent interactions when protein residues become (partially) buried at the dry contact region. Values of 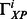 for residues at the contact region of D44.1 and lysozyme in the free states are balanced (Figure S3), such that the combined contribution of contact residues to the protein-associated change of 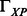 is negligible

**Figure 7:**
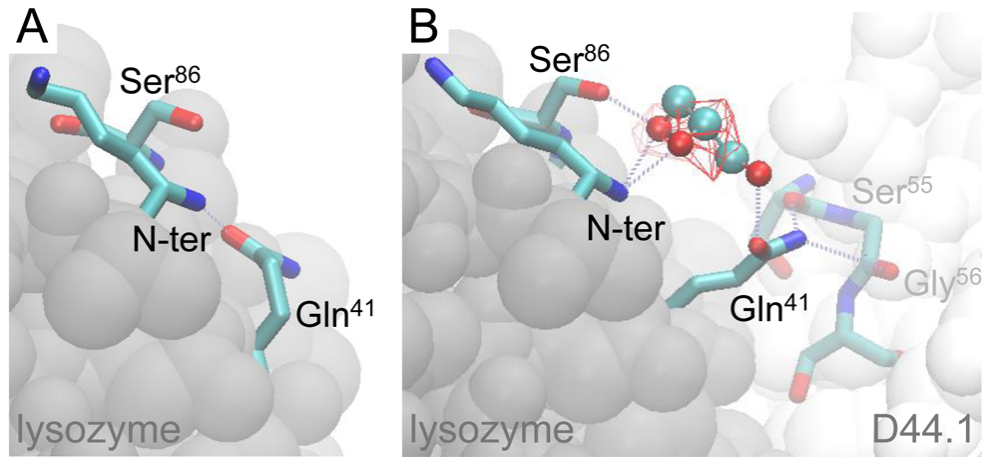
Snapshot of glycerol molecules near the N-terminus of lysozyme in the free state (A) and associated to D44.1 (B). Hydrogen-bonds are indicated by dotted lines and red wireframes demark solvent regions with high local glycerol concentrations (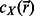
>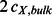).

Another distinctive feature of the D44.1-lysozyme complex is that several residues with significant changes of 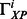 are located further from the protein-protein contact region (Figure 4 and Figure S3). Closer examination of local protein solvation near these residues reveals that changes of 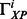 are caused by the specific rearrangement of protein side-chains upon protein-protein association. This is illustrated for the protein surface region near the N-terminus of lysozyme, which is preferentially hydrated in the free state, but becomes preferentially solvated by glycerol in the associated state (Figure 5 and Figure S3). In the free state of lysozyme, Gln^41^ forms intramolecular hydrogen-bonds with adjacent residues including the N-terminus (Figure 7A), but in the D44.1-lysozyme complex, Gln^41^ adopts extended orientations as it forms hydrogen-bonds with D44.1 (Figure 7B). Extended orientations of Gln^41^ favor the formation of multiple hydrogen-bonds between glycerol and several lysozyme-residues including Gln^41^, Ser^86^ and the N-terminus (Figure 7B and Movie S1). This leads to strong preferential solvation of the corresponding protein locus in the D44.1-lysozyme complex.

## Discussion

In this study, we have characterized the opposite effects of glycerol on the association constants of two antibodies against lysozyme using surface plasmon resonance, and we have used molecular dynamics simulations to quantify preferential interaction coefficients of the corresponding proteins in the free and associated states. Our results indicate that glycerol weakens the association of D1.3 with lysozyme because of the overall decrease in preferential interactions as a result of the total exclusion of glycerol, but not of water, from the protein-protein contact region (Table 1, Figure 4 and Figure 5). Conversely, glycerol strengthens the association of D44.1 with lysozyme because of the overall increase in preferential interactions due to (1) exclusion of water from the dry protein-protein contact region (Figure 5) and (2) rearrangement of specific protein side-chains at the periphery of the D44.1-lysozyme interface resulting in local preferential binding of glycerol through multiple hydrogen-bonding (Figure 7). These results demonstrate the direct relationship between macroscopic solvent effects on protein-protein interactions and atom-scale solvent-protein interactions, and show that cosolvent-effects on protein-protein interactions critically depend on the extent of dewetting of the protein-protein contact region and on local protein structural changes that alter cooperative solvent interactions with adjacent residues.

Our surface plasmon resonance data showed that the association constants of both antibodies change exponentially with glycerol molality over the entire concentration range investigated (0–9 molal glycerol) (Figure 1). Exponential responses of equilibrium constants K_A_ with respect to cosolvent molality have been observed for many biomolecular reactions [21], [43]–[54], and it has been suggested that the underlying mechanisms are closely related [17]. Considering the direct relationship between solvent-protein interactions and solvent effects on protein reactions (Eq. 1 and Eq. 2), exponential responses of K_A_ can be attributed to the linear behavior of 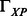 with respect to cosolvent molality. Linear behavior of 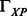 with respect to cosolvent molality has been observed for a wide range of proteins and cosolvents [55]–[58], and can be explained by considering solvent exchange equilibria at protein surface sites that weakly interact with solvent molecules [59]. Taken together, these points support the notion that exponential responses of biomolecular equilibria with respect to cosolvent molality reflect linear changes of 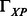 caused by differences in weak solvent-protein interactions between different biomolecular states.

Our methodology for quantifying the molecular origins of solvent effects on protein-protein interactions comprises the following steps: (1) run extended molecular dynamics simulations of free and associated proteins with constrained backbone coordinates, (2) calculate global, regional and residue-based preferential interaction coefficients and local concentration maps of free and associated proteins, (3) determine the protein-protein interface region inte(D) where protein solvation changes occur, (4) quantify cosolvent effects on the protein-protein association constant K_A_ from regional preferential interaction coefficients at the interface region inte(D), (5) identify and map protein residues for which residue-based preferential interaction coefficients significantly differ between associated and free proteins, (6) analyze local solvation changes near these residues by inspecting local concentration maps and solvent trajectories. We found that Step 3 of our methodology is critical as it enables the calculation of protein association-induced changes of preferential interaction coefficients with high precision (Table 1). Such high precision is needed for Step 4, and can generally not be obtained from experiment [56], [57]. Another important feature of our methodology is the identification of specific loci at the protein surface that contribute to macroscopic solvent effects on protein-protein interactions (Step 5). This enables the user to locate and quantify local solvation changes that determine macroscopic solvent effects on protein-protein interactions.

In a previous molecular dynamics study with unconstrained protein coordinates, we found that large conformational changes of the protein backbone result in large changes of the preferential interaction coefficient 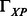 [58]. Trajectory-dependent sampling of the protein conformational ensemble caused large differences of 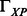-values obtained from independent simulations, and 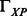-values of specific protein conformations sampled within nanoseconds differed by several units [58]. Such large differences of 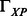 are of similar magnitude as the differences of 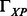 between free and associated proteins (Table 1), and differentiating protein-association induced changes of 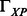 from trajectory-dependent conformational sampling effects would be extremely challenging. Moreover, quantitative characterization of local protein solvation is currently only possible for simulations with constrained backbone coordinates [40]. Constraining backbone coordinates is therefore an essential feature of our methodology. An arguable limitation of using constrained backbone coordinates is that protein-association induced conformational changes of the protein backbone that could significantly affect solvent preferential interactions are not accounted for. However, such conformational changes are expected to be rare since backbone conformations for most protein complexes differ little between the free and associated protein states [60], [61].

Owing to the important role of water in protein binding [62]–[70], much recent research effort has evolved in fairly accurate methods for predicting the location of crystallographically observed waters at the interface of protein cavities and small molecule ligands [65], [71]–[74]. Hydration sites at protein-protein interfaces may be more difficult to predict, and studies on hydration of protein-protein interfaces have been mainly limited to the analysis of crystal waters [75]–[78]. In this study, we obtained good agreement between the location of high-occupancy water sites and crystal waters at the protein-protein interface (Figure S4). Over the course of the simulation, all water molecules at the protein-protein interface undergo dynamic interchange between different solvation sites (Movie S2), and the protein-protein interface region contacts many more water molecules than the waters resolved in the crystal structure (Figure S4). All these waters contribute to the overall preferential interaction coefficient, and it is therefore not surprising that crystallographic studies of protein solvation fail to explain cosolvent effects on protein-protein interactions [79].

To this day, cosolvent effects on protein reactions are commonly interpreted based on the global preferential interaction coefficient of the native free protein state and the change of surface area involved in the reaction [6], [56]. Thereby, it is – often implicitly - assumed that local protein solvation is homogeneous over the entire protein surface. Based on this assumption, one would conclude that glycerol – which is, on average, preferentially excluded from the protein surface – would always strengthen protein-protein interactions. The flaw of the underlying assumption is evidenced by our results which reveal a remarkable heterogeneity of differences between local preferential solvent interactions in the free and associated protein states (Figure 4 and Figure S2). A more detailed approach for predicting solvent effects on protein reactions was pioneered by Tanford, who quantified thermodynamic solvent effects on smaller constituent groups of a protein molecule and hypothesized the additivity of individual contributions of the constituent groups [80]. Group transfer models, however, cannot account for hydration changes at the protein-protein contact regions and cooperative interactions of cosolvent molecules with adjacent protein residues. We find that these features play a key role in determining solvent effects on protein-protein interactions, and we conclude that quantitative characterization of local protein solvation is prerequisite for understanding cosolvent effects on protein-protein interactions.

Quantitative characterization of local protein solvation requires atomic protein structures, accurate force fields and computational resources for running long protein simulations (>100 ns) [40]. Atomic protein structures can be retrieved from the Protein Data Bank (PDB) which covers more than 25% of the human genome and includes more than 10,000 protein complexes [81], [82]. Force fields validated against experimental values of protein preferential interaction coefficients are available for several cosolvents [42], [58], [83], and future research is expected to increase this list. Computational resources for running long all-atom simulations of large protein complexes may appear daunting at first sight. However, since protein-protein association only affects solvation near the protein-protein interface (Figure 1 and Table 1), computational costs could be significantly reduced by truncating the simulation system around the protein-protein interface region. In this way, sufficiently long simulations may be achieved using standard high performance clusters.

Granted the availability of accurate force fields, our methodology may also be used to study crowding effects on protein association. Similar to small-molecule cosolvents, effects of macromolecular crowders on protein association are protein-dependent [84] and appear to be the balanced result of steric exclusion and specific crowder-protein interactions [23], [85], [86]. By including chemical details of the protein and the macromolecular crowder, our methodology could significantly improve current crowding models which generally fail to quantitatively reproduce crowding effects on protein association [87]. Finally, we would like to point out that the scope of our methodology is not restricted to protein-protein interactions, but extends to any molecular recognition process that involves the formation of supramolecular complexes with well-defined atomic structures. Our methodology may therefore prove an important tool to elucidate solvent effects on molecular recognition processes and protein function in diverse biological environments.

## Materials and Methods

### Protein expression and purification

The genes of scFv D1.3 and Fab D44.1 were cloned into pET-39b(+) vectors (Novagen) and expressed in E. Coli BL21(DE3). scFv D1.3 was recovered from the periplasmic fraction by osmotic shock, and Fab D44.1 was refolded from the insoluble cell fraction. The recombinant proteins were purified by affinity chromatography using CnBr-Sepharose FF resin (GE Healthcare) coupled to lysozyme. The purity of the proteins was estimated to be >95% as judged by SDS-PAGE. Protein concentrations were calculated using a UV_280 nm_ absorption coefficient (mL.mg^−1^.cm^−1^) of 1.80 for scFv D1.3 and 1.60 for Fab D44.1. Further details are described in Text S1.

### Surface plasmon resonance

The effects of glycerol on the binding affinity of scFv D1.3 and Fab D44.1 with lysozyme were measured by surface plasmon resonance using a BIACORE 3000 system (GE Healthcare). Lysozyme was coupled to a CM5 sensor chip (GE Healthcare) using amine coupling. Antibody fragments were diluted in buffer with 0–9 molal glycerol to concentrations ranging from 10–2000 nM, and injected into the sensor chip for 7.5 minutes. Associated antibody fragments were subsequently dissociated by flowing buffer with 0–9 molal over the chip for 8 minutes. The chip was then regenerated by injecting 10 mM HCl for 30 seconds. For each glycerol concentration, association constants (K_A_) were determined from Scatchard analysis by measuring steady-state-responses at 6 different protein concentrations. Further details are described in Text S1.

### Molecular dynamics simulations

Six independent molecular dynamics simulations were run for Fv D1.3, Fab D44.1 and lysozyme in the free and associated states in a 6 molal aqueous solution of glycerol. Protein structures for the D1.3-lysozyme and D44.1-lysozyme complexes were retrieved from PDB-structures 1VFB [63] and 1MLC [88], respectively, and crystal waters at the protein-protein interface were included in the starting structures of the associated states. For all simulations, a minimum of 10 Å between the protein and the boundary of the solvent box was kept. The CHARMM22 parameter set [89] was used to model protein atoms, water was modeled by the TIP3-model [90] and force field parameters for glycerol were taken from the carbohydrate hydrate parameters developed by Liang and Brady (the parameters are available at http://mackerell.umaryland.edu/CHARMM_ff_params.html under the link toppar_c32b1.tar.gz in the file par_all22_sugar.inp) with partial charges published by Reiling et al. [91]. Simulations were run with NAMD v2.7 [92] with constrained protein backbone coordinates for at least 160 ns, which is longer than the minimum simulation time for characterizing local protein solvation in mixed solvents [40]. Further details are described in Text S1.

### Characterization of local protein solvation

Local protein solvation of D1.3, D44.1 and lysozyme in the free and associated states was analyzed from the respective MD simulations following a newly developed method for quantitative characterization of local protein solvation [40]. Local concentrations were calculated based on the solvent occupancy of a three-dimensional grid and visualized with the software VMD 1.9 [93]. Global preferential interaction coefficients 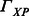, residue-based preferential interactions coefficients 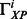, and regional preferential interactions coefficients 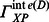 and 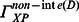, were calculated from the average number of water and glycerol molecules within 5 Å from the corresponding protein van der Waals surfaces [40], [94]. Standard errors of preferential interaction coefficients were calculated by dividing the simulation trajectories in time blocks of increasing length followed by systematic analysis of the corresponding standard deviations [94]. Further details are described in Text S1.

## Supporting Information

Figure S1 Local concentration maps of the associated and free proteins of the D44.1-lysozyme complex. Solvent regions that are preferentially solvated by glycerol or water are colored red and blue respectively, and solvent regions near the interface region are highlighted.
(TIF)

Figure S2Local preferential interaction coefficients 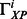 of interface residues of free (green squares) and associated (blue diamonds) proteins of the D1.3-lysozyme complex. Interface residues are indicated by grey bars on the X-axis, and 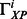-values are only depicted for residues for which 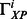 significantly differs between free and associated proteins. 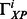-values corresponding with Asp^54^ of D1.3 V_H_ in the free and associated states are indicated by red arrows.(TIF)

Figure S3Local preferential interaction coefficients 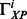 of interface residues of free and associated proteins for the lysozyme-D44.1 complex. Interface residues are indicated by grey bars on the X-axis, and 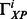-values are only depicted for residues for which 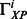 significantly differs between free and associated proteins.(TIF)

Figure S4 Hydration at the protein-protein interface in the D1.3-lysozyme complex. A) Waters resolved in the crystal structure. B) Snapshot of interface waters after 100 ns of simulation. C) Local concentration map of water calculated from the entire simulation.
(TIF)

Movie S1Local protein solvation by glycerol molecules near the N-terminus of lysozyme in the D44.1-lysozyme complex. Image frames were rendered every 1 ns with VMD 1.9 [93] and include glycerol molecules with at least one atom within 4 Å of the N-terminus and Gln^41^ of lysozyme. The red wireframe demarks a solvent region with high local glycerol concentration (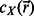
>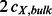). Note that this region is occupied by glycerol more than half the time.(AVI)

Movie S2 Hydration of the protein-protein interface region of lysozyme in the D1.3-lysozyme complex. Out of 48 crystal waters, only three crystal waters remain at the protein-protein interface during the entire simulation (represented as colored spheres). All other water molecules within 4 Å of the protein-protein interface region are represented as blue spheres.
(AVI)

Text S1 Detailed description of experimental and computational methods, and derivation of thermodynamic equations.
(DOC)
