# Supplementary figures and images for: Quantifying the Molecular Origins of Opposite Solvent Effects on Protein-Protein Interactions
Source: PLoS Comput Biol. 2013 May 16;9(5):e1003072. doi: 10.1371/journal.pcbi.1003072 (PMC3656110; doi:10.1371/journal.pcbi.1003072)

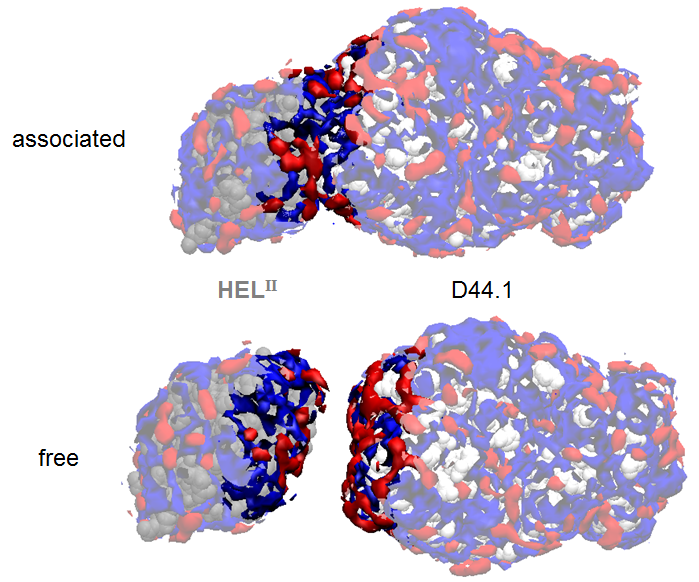

Supplement: Figure S1 — Local concentration maps of the associated and free proteins of the D44.1-lysozyme complex. Solvent regions that are preferentially solvated by glycerol or water are colored red and blue respectively, and solvent regions near the interface region are highlighted. (TIF) [file pcbi.1003072.s001.tif]

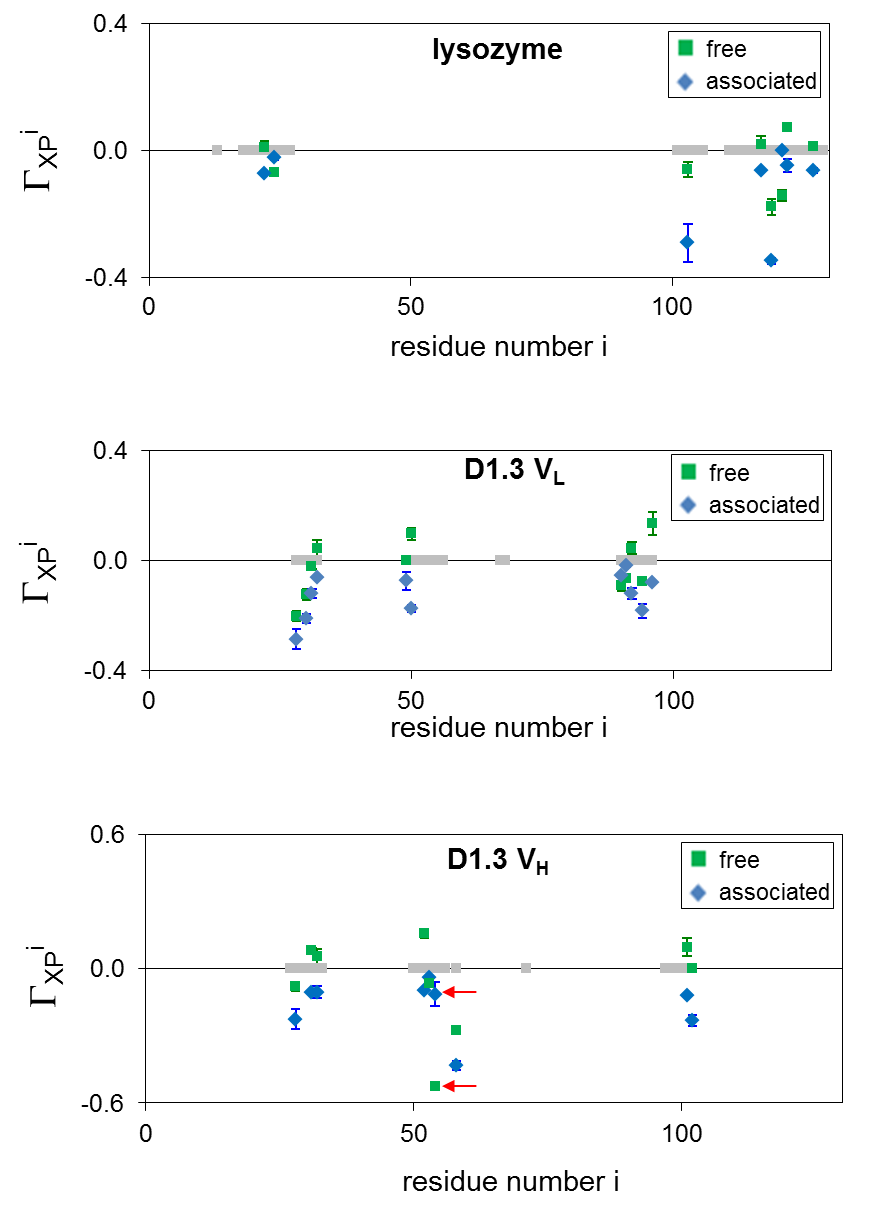

Supplement: Figure S2 — Local preferential interaction coefficients of interface residues of free (green squares) and associated (blue diamonds) proteins of the D1.3-lysozyme complex. Interface residues are indicated by grey bars on the X-axis, and -values are only depicted for residues for which significantly differs between free and associated proteins. -values corresponding with Asp54 of D1.3 VH in the free and associated states are indicated by red arrows. (TIF) [file pcbi.1003072.s002.tif]

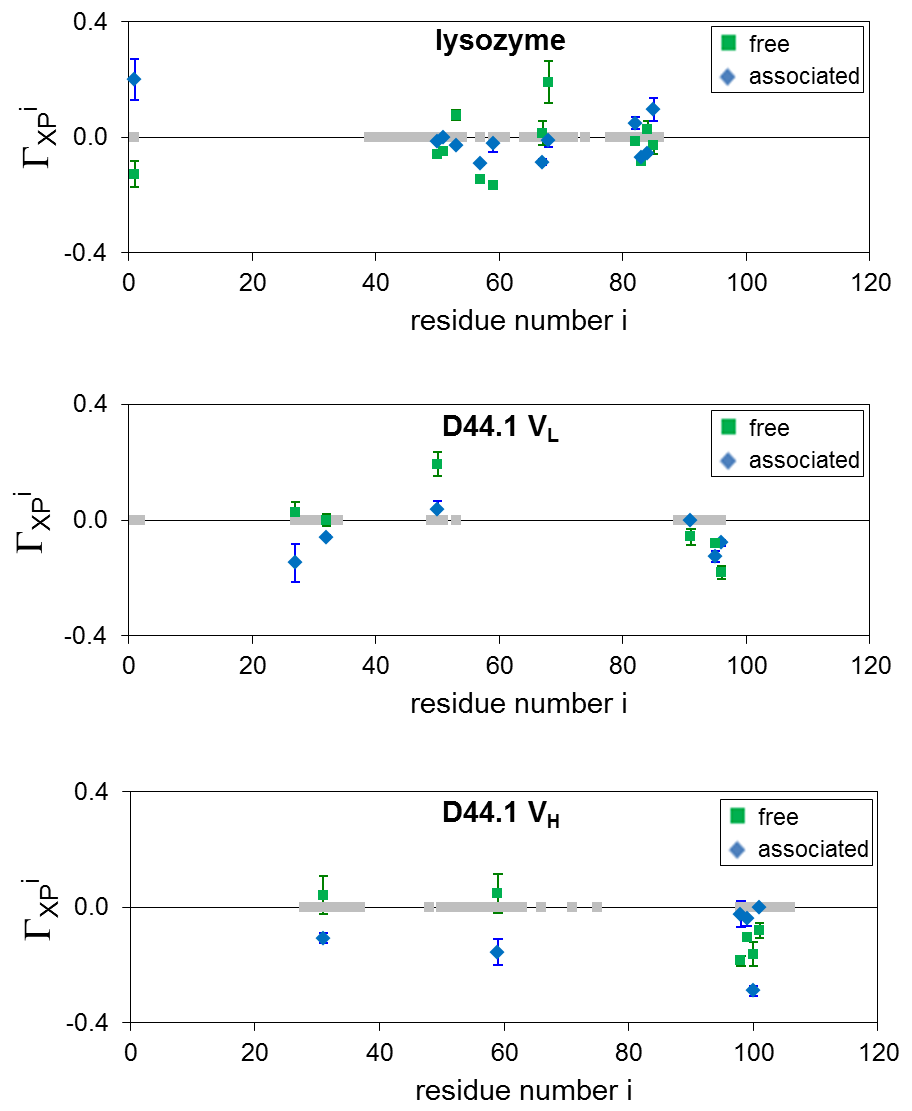

Supplement: Figure S3 — Local preferential interaction coefficients of interface residues of free and associated proteins for the lysozyme-D44.1 complex. Interface residues are indicated by grey bars on the X-axis, and -values are only depicted for residues for which significantly differs between free and associated proteins. (TIF) [file pcbi.1003072.s003.tif]

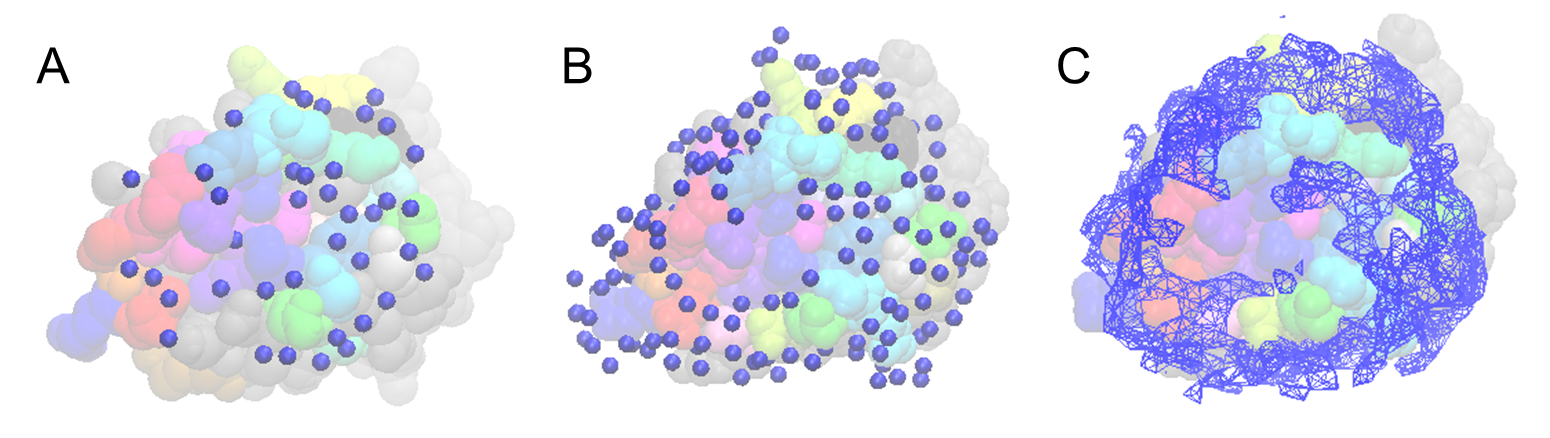

Supplement: Figure S4 — Hydration at the protein-protein interface in the D1.3-lysozyme complex. A) Waters resolved in the crystal structure. B) Snapshot of interface waters after 100 ns of simulation. C) Local concentration map of water calculated from the entire simulation. (TIF) [file pcbi.1003072.s004.tif]
