# Supplementary material for: Quantifying the Molecular Origins of Opposite Solvent Effects on Protein-Protein Interactions
Source: PLoS Comput Biol. 2013 May 16;9(5):e1003072. doi: 10.1371/journal.pcbi.1003072 (PMC3656110; doi:10.1371/journal.pcbi.1003072)
Supplement: Text S1 — Detailed description of experimental and computational methods, and derivation of thermodynamic equations. (DOC) [file pcbi.1003072.s007.doc]

# Quantifying the molecular origins of opposite solvent effects on protein-protein interactions

# Supporting Information

[[1]](#footnote-2) Vincent Vagenende*, Alvin X. Han, Han B. Pek and Bernard L.W. Loo

Bioprocessing Technology Institute, A*STAR (Agency for Science, Technology and Research), 20 Biopolis Way #06-01 Centros, Singapore 138668

Supporting Methods

## Protein Expression and Purification

The VH and VL domains of the antibody D.13 were cloned as a single chain in a pET-39b(+) vector (Novagen) and transfected into competent Escherichia Coli BL21(DE3) cells. Cells were grown in a shake-flask at 30 °C in 2x yeast extract tryptone medium to OD600nm  of ~ 1.0 AU, followed by addition of IPTG (20 μM) to induce the expression of D1.3. The fermentation was continued 4 hours following induction, and the fermentation broth was centrifuged at 4,000 rpm for 20 minutes. Proteins were recovered from the periplasm by osmotic shock.

The Fab fragment of antibody D44.1 was cloned into the pET-39b(+) vector (Novagen) and transfected into competent Escherichia coli BL21(DE3) cells. Fermentation and induction conditions were identical to the expression of scFv D1.3. Analysis of the expression levels by Western blot showed that the expressed Fab D44.1 was mostly insoluble. To recover protein from inclusion bodies, the fermentation broth was centrifuged (4,000 rpm, 20 min) and the cell pellet was re-suspended in 100 mL of 20 mM EDTA, 50 mM Tris (pH 7.8) and homogenized at 10,000 psi by passing it twice through a Microfluidizer (M-110P, Microfluidics). The homogenate was then centrifuged (17,000 rpm, 15 min) and the pellet was re-suspended in 100 mL of 20 mM EDTA, 0.02% Tween, 50 mM Tris (pH 7.8). The suspension was centrifuged (17,000 rpm, 15 min) and the pellet was denatured in 10 mL of denaturing buffer (6 M Guanidine-HCl, 100 mM Arginine, 50 mM NaCl, 100 mM DTT, and 100mM Tris, pH 8.0). The total protein content was determined by Bradford analysis and the protein solution was diluted in denaturing buffer to 2 mg/mL prior to refolding. To refold Fab D44.1, the denatured protein solution was diluted 40 times with an optimized refolding buffer (100 mM Arginine, 50 mM NaCl, 1.44 M glycerol, 3.4 mM oxidized glutathione, 100 mM Tris, pH 8.0). The protein solution was incubated in the refolding buffer for at least 48 hours prior to further purification.

Fab D44.1 and scFv D1.3 were purified by affinity chromatography using CnBr-Sepharose FF resin (GE Healthcare) coupled to Hen Egg-white Lysozyme (Sigma). Coupled resin (1 mL) was packed in a 5/50 Tricorn column (GE Healthcare), and protein-containing fractions were loaded using an AKTA Explorer system (GE Healthcare). Proteins were eluted at pH 2.5, and directly neutralized to pH 7.0 by adding 1M Tris-HCl (pH 8.0). The purity of the proteins was estimated to be > 95% as judged by SDS-PAGE. Protein concentrations were calculated using a UV280nm­ absorption coefficient (mL.mg-1.cm-1 ) of 1.80 for scFv D1.3 and 1.60 for Fab D44.1. Purified proteins were concentrated to 0.1 – 0.3 mg/mL using centrifugal concentrators (Vivaspin PES MWCO 10000, Sartorius Stedim).

## Surface Plasmon Resonance

To characterize the effect of glycerol on the binding affinity of scFv D1.3 and Fab D44.1, surface plasmon resonance (SPR) experiments were carried out on a BIACORE 3000 system (GE Healthcare). Lysozyme was coupled to a CM5 sensor chip (GE Healthcare) using the amine coupling kit (GE Healthcare), and the sensor chip was primed with HBS buffer (10 mM HEPES, pH 7.4, 150 mM NaCl, 3.4 mM EDTA, 0.005% v/v Surfactant P20) with the specified concentration of glycerol (0, 3, 5.9 and 9 molal) prior to each experiment. Protein samples were extensively buffer-exchanged in the respective HBS-glycerol buffers using centrifugal concentrators (Vivaspin PES MWCO 10,000, Sartorius Stedim). For one sample condition – i.e. D44.1 at 9 molal glycerol – we noticed extensive protein precipitation; hence, no further measurements were carried out for this sample. Protein samples were diluted to concentrations ranging from 10 – 2000 nM, and injected into the sensor chip for 7.5 minutes at 5 μL/min (association phase). Proteins were subsequently dissociated by flowing HBS-glycerol buffer over the chip for 8 minutes (dissociation phase). Finally, the chip was regenerated by injecting 10 mM HCl for 30 seconds. For each glycerol concentration, association constants (KA) were determined from Scatchard analysis by measuring steady-state-responses at 6 different protein concentrations . Standard deviations were determined from two independent determinations and the ratio of the association constants (KA/KA,0) was obtained by dividing the average values of the association constant with and without glycerol.

Association constants were also determined from kinetic analysis by fitting sensorgrams to a 1:1 binding model (BIAevaluation software, GE Healthcare). The association rate constant ka and the dissociation rate constant kd at different glycerol concentrations were obtained from the respective fits, and the equilibrium association constant KA was calculated as the ratio ka/kd. The ratio of the association constants with and without glycerol (KA/KA,0) determined from kinetic analysis agreed very well with the values obtained from Scatchard analysis.

## Thermodynamic Equations

### Linked functions and preferential interactions

The equilibrium of two molecules A and B in the free and associated state is characterized by the association constant KA :

$K_{A}=\frac{[AB]}{[A].[B]}$ (S1)

The association constant KA generally depends on solvent conditions, such as pH and the presence of other solutes. Several decades ago, Wyman quantified the change of KA when adding a ligand X through the so-called Wyman linkage function:

$\frac{\delta\ln K_{A}}{\delta\ln a_{X}}=\nu_{X}^{AB}-\nu_{X}^{A}-\nu_{X}^{B}=\Delta\nu_{X}$ (S2)

With aX the activity of ligand X and $\nu_{X}^{AB}$,$\nu_{X}^{A}$ and $\nu_{X}^{B}$ the numbers of ligand molecules bound to the associated state AB and the free molecules A and B, respectively. Tanford later generalized Wyman’s linkage function for weakly binding solutes by accounting for changes of the number of bound solute and water molecules that accompany the association reaction:

$\frac{\delta\ln K_{A}}{\delta\ln a_{X}}=\Delta\nu_{X}-\frac{m_{X}}{m_{W}}\Delta\nu_{W}$ (S3)

In the above equation, mX and mW­ are the molalities of solute (subscript X) and water (subscript W), and $\Delta\nu_{X}$ ($\Delta\nu_{W}$) are the differences in the number of solute (water) molecules that bind to the associated state AB compared to the free states A and B. From Eq. S3 it is seen that for solutes that strongly bind at low molality the effect of hydration changes becomes negligible. In that case Eq. S3 becomes identical to the original Wyman linkage function (Eq. S2). In contrast, solutes that bind to A, B and AB with similar affinities as water only affect the association reaction if they are present at sufficiently high concentrations. Such weakly interacting solutes are often referred to as cosolvents and comprise a wide class of molecules including osmolytes, denaturants and salts .

Strong binding of solutes to a molecule can be characterized by experimental techniques such as X-ray crystallography or NMR, and determining the number of strongly bound solutes per molecule is relatively straightforward. Not so for weakly interacting solutes. Indeed, the average number of cosolvent and water molecules associated with a certain molecule is determined by complex solvation properties resulting from the continuous motion of cosolvent and water at the molecular surface . Small preferences of surface loci for cosolvent or water result in local enrichment of water (preferential hydration) or cosolvent (preferential solvation) , and the extent of enrichment averaged over entire the surface a molecule is quantified by the preferential interaction coefficient $\Gamma_{XP}$ :

$\Gamma_{X}^{ } = n_{X}(r<R) - \frac{m_{X}}{m_{W}} n_{W}(r<R)$ (S4)

In the above equation nX­ and nW are the average number of cosolvent and water molecules within a radius R of the molecular surface where preferential interactions occur. Wyman’s modified linkage function can thus be expressed in terms of preferential interaction coefficients :

$\frac{\delta\ln K_{A}}{\delta\ln a_{X}}=\Delta\Gamma_{XP}$ (S5)

In this equation, $\Delta\Gamma_{XP}$ is the difference of the preferential interaction coefficients of A and B in the associated state and the free state, i.e. $\Delta\Gamma_{XP}=\Gamma_{XP}^{AB}-\Gamma_{XP}^{A}-\Gamma_{XP}^{B}$. Equation S5 can be considered as a generalization of the original linkage function presented by Wyman (Eq. S2) and can be directly derived from the thermodynamical theory of 3-component solutions .

### Molecular association in aqueous glycerol solutions

Equation S5 is generally applicable for quantifying cosolvent effects on the association of two molecules, regardless of the type of cosolvent. For molecular association in aqueous glycerol solution, Eq. S5 can be further simplified by taking into account activity data of aqueous glycerol solutions :

$\frac{\delta\ln K_{A}}{\delta m_{X}}=\frac{\Delta\Gamma_{XP}}{m_{X}}$ (S6)

Preferential interaction coefficients of glycerol with proteins are generally linear with respect to glycerol molality , and linearity of $\Gamma_{XP}$ with glycerol molality has also been approximately established in previous simulations . Linearity of $\Gamma_{XP}$ results in linearity of KA, and it follows from Eq. S6 that the effects of glycerol on KA are determined over the entire concentration range (0 – 9 molal glycerol) from $\Gamma_{XP}$-values at any single glycerol molality $m_{X}^{S}$ :

$\Delta\ln K_{A,m_{x}}=\frac{\Delta\Gamma_{XP,m_{X}^{S}}}{m_{X}^{S}}m_{X}$ (S7)

When glycerol molalities $m_{X}$and $m_{X}^{S}$ are equal, we can further simply Equation S7:

$\Delta\ln K_{A}=\Delta\Gamma_{XP}$ (S8)

Equation S8 specifies that the difference of the logarithms of the association constants KA in a glycerol-water mixture and in pure water equals the difference of $\Gamma_{XP}^{ }$ between the associated and free protein states in the glycerol-water mixture.

## Molecular Dynamics Simulations

Six independent molecular dynamics simulations were run for Fv D1.3, Fab D44.1 and lysozyme in the free and associated states in an aqueous solution with 6 molal glycerol (Table T1). Protein structures for the D1.3-lysozyme and D44.1-lysozyme complexes were retrieved from PDB-structures 1VFB and 1MLC , respectively, and crystal waters at the protein-protein interface were included in the starting structures of the associated states. Charges of Arg, Lys and the N-terminus were set positive, charges for Asp, Glu and the C-terminus were set negative, and all other amino acids were neutral. Proteins in the free and associated states were solvated in water with 6 molal glycerol and the protein charges were neutralized by either Cl-atoms or Na-atoms. The total number of water (nw) and cosolvent (nx) molecules in the solvent boxes for the respective simulations are listed in (Table T1). For all simulations, a minimum of 10 Å between the protein and the boundary of the solvent box was kept. The CHARMM22 parameter set was used to model protein atoms and water was modeled by the TIP3-model . Similar to previous studies , we used force field parameters for glycerol which are based on the carbohydrate hydrate parameters developed by Liang and Brady (these parameters are available on the website of Alexander Mackerell at http://mackerell.umaryland.edu/CHARMM_ff_params.html under the folder [toppar_c32b1.tar.gz](http://mackerell.umaryland.edu/download.php?filename=CHARMM_ff_params_files/toppar_c32b1.tar.gz) in the file par_all22_sugar.inp) with partial charges of glycerol published by Reiling et al. .

Table T1: Simulation details

| Protein | PDB  structure | Time  [ns] | nwa | nxa |
| --- | --- | --- | --- | --- |
|
| lysozyme | 1VFB | 1200 | 4862 | 514 |
| D1.3 | 1VFB | 1200 | 6861 | 725 |
| D1.3-lysozyme | 1VFB | 160 | 11265 | 1190 |
| lysozyme | 1MLC | 160 | 5064 | 535 |
| D44.1 | 1MLC | 160 | 10663 | 1127 |
| D44.1-lysozyme | 1MLC | 160 | 14573 | 1540 |
| a nw and nx are the total numbers of water and glycerol molecules in the simulated systems | | | | |

Each of the six solvated protein systems was minimized for 1000 steps in NAMD v2.7 and simulations were run in the NpT ensemble (1 atm, 298 K) using a 2 fs step size while keeping the bond lengths of hydrogen atoms constant with the SHAKE algorithm. The system temperature was controlled using Langevin dynamics with a coupling coefficient of 5 ps-1. The system pressure was controlled using Langevin piston pressure control with a period of 100 fs and a barostat damping timescale of 50 fs. For all simulations, periodic boundary conditions were used, and images and non-bonded lists were updated every 10 steps using a 12 Å cutoff distance. The van der Waals potential energy was smoothly switched off between 8 and 10 Å, and the particle-mesh Ewald method was used to calculate long-range electrostatic interactions. All systems were simulated for at least 160 ns (Table T1), which is longer than the minimum simulation time for characterizing local protein solvation in mixed solvents .

In a previous study, we found that that conformational changes of the protein backbone in the first nanoseconds of MD simulations with unconstrained protein coordinates result in relatively large changes of the preferential interaction coefficient $\Gamma_{XP}$ . Another study with constrained back-bone coordinates pointed out that, unlike differences of $\Gamma_{XP}$ between distinct backbone conformations, differences of $\Gamma_{XP}$ between distinct side-chains are generally small or non-existent . Hence, we chose to run simulations with constrained backbone coordinates as such simulation conditions prevent large conformational changes but account for local protein dynamics.

## Characterization of Local Protein Solvation

Local protein solvation of D1.3, D44.1 and lysozyme in the free and associated states was analyzed from the respective MD simulations following our newly developed methodology for quantitative characterization of local protein solvation . Our analysis includes computation of regional and residue-based preferential interaction coefficients, visual inspection of local concentration maps, and the analysis of the trajectories of specific solvent molecules and protein surface loci. In addition, we mapped differences of residue-based preferential interaction coefficients between the free and associated protein states using the software VMD .

### Preferential interaction coefficients

Global preferential interaction coefficients $\Gamma_{XP}$ and residue-based preferential interactions coefficients $\Gamma_{XP}^{i}$ were calculated by counting water and cosolvent molecules within 5 Å from the protein van der Waals surface as described previously :

$\Gamma_{XP}^{ } = \left\langle n_{XP}(r<5Å) - \frac{n_{X}-n_{XP}(r<5Å)}{n_{W}-n_{WP}(r<5Å)} n_{WP}(r<5Å)\right\rangle_{\tau_{run}}$ (S9)

$\Gamma_{XP}^{ i} = \left\langle n_{XP}^{i}(r<5Å) - \frac{n_{X}-n_{XP}(r<5Å)}{n_{W}-n_{WP}(r<5Å)} n_{WP}^{i}(r<5Å)\right\rangle_{\tau_{run}}$ (S10)

In the above equations, $n_{X}$ and $n_{W}$ are the total number of cosolvent and water molecules in the simulation box, $n_{XP}(r<5Å)$ and $n_{WP}(r<5Å)$ are the total number of cosolvent and water molecules for which the center of mass is within 5 $\text{Å}$ from the protein van der Waals surface, and $n_{XP}^{i}(r<5Å)$ and $n_{WP}^{i}(r<5Å)$ are the number of cosolvent and water molecules within 5 $\text{Å}$ from the protein surface that are closer to protein residue *i* than to any other residue*.* Brackets refer to the time average over the entire simulation time $\tau_{run}$.

Regional preferential interaction coefficients for the protein-protein interface region inte(D) and the complementary region non-inte(D), respectively, were calculated as follows:

$\Gamma_{XP}^{\mathrm{int}e(D)} = \left\langle\sum n_{XP}^{\mathrm{int}e(D)}(r<5Å) - \frac{n_{X}-n_{XP}(r<5Å)}{n_{W}-n_{WP}(r<5Å)} \sum n_{WP}^{\mathrm{int}e(D)}(r<5Å)\right\rangle_{\tau_{run}}$ (S11)

$\Gamma_{XP}^{not-\mathrm{int}e(D)} = \left\langle\sum n_{XP}^{not-\mathrm{int}e(D)}(r<5Å) - \frac{n_{X}-n_{XP}(r<5Å)}{n_{W}-n_{WP}(r<5Å)} \sum n_{WP}^{not-\mathrm{int}e(D)}(r<5Å)\right\rangle_{\tau_{run}}$ (S12)

In the above equations,$n_{XP}^{\mathrm{int}e(D)}(r<5Å)$and $n_{XP}^{not-\mathrm{int}e(D)}(r<5Å)$ are the number of solvent molecules within 5 Å from the protein van der Waals surface for which the closest protein atom belong to inte(D) and non-inte(D), respectively.

### Local concentration maps

Local concentrations were calculated based on the solvent occupancy of a three-dimensional grid with grid size 1.5 Å and visualized with the software VMD 1.9 , as described previously in Vagenende et al. . Unless indicated otherwise, cut-off values were selected such that local concentration maps depict all solvent regions with local solvent concentrations $c_{\alpha}(\vec{r})$ greater than the respective bulk solvent concentrations $c_{\alpha,bulk}$.

Supporting References

1. McCafferty J, Griffiths AD, Winter G, Chiswell DJ (1990) PHAGE ANTIBODIES - FILAMENTOUS PHAGE DISPLAYING ANTIBODY VARIABLE DOMAINS. Nature 348: 552-554.

2. Braden BC, Souchon H, Eisele JL, Bentley GA, Bhat TN, et al. (1994) 3-dimensional structures of the free and the antigen-complex Fab from monoclonal antilysozyme antibody - D44.1. Journal of Molecular Biology 243: 767-781.

3. Dall'Acqua W, Goldman ER, Eisenstein E, Mariuzza RA (1996) A mutational analysis of the binding of two different proteins to the same antibody. Biochemistry 35: 9667-9676.

4. Wyman J (1964) Linked functions and reciprocal effect in hemoglobin: a second look. Advances in Protein Chemistry 19: 223-286.

5. Timasheff SN (1998) Control of protein stability and reactions by weakly interacting cosolvents: The simplicity of the complicated. Advances in Protein Chemistry 51: 355-432.

6. Schellman JA (2003) Protein stability in mixed solvents: A balance of contact interaction and excluded volume. Biophysical Journal 85: 108-125.

7. Vagenende V, Trout BL (2012) Quantitative characterization of local protein solvation to predict solvent effects on protein structure. Biophysical Journal 103: 1354–1362.

8. Shimizu S (2004a) Estimating hydration changes upon biomolecular reactions from osmotic stress, high pressure, and preferential hydration experiments. Proceedings of the National Academy of Sciences of the United States of America 101: 1195-1199.

9. Smith PE (2004) Local chemical potential equalization model for cosolvent effects on biomolecular equilibria. Journal of Physical Chemistry B 108: 16271-16278.

10. Record MT, Zhang WT, Anderson CF (1998) Analysis of effects of salts and uncharged solutes on protein and nucleic acid equilibria and processes: A practical guide to recognizing and interpreting polyelectrolyte effects, Hofmeister effects, and osmotic effects of salts. Advances in Protein Chemistry 51: 281-353.

11. Scatchard G, Hamer WJ, Wood SE (1938) Isotonic solutions. I. The chemical potential of water in aqueous solutions of sodium chloride, potassium chloride, sulfuric acid, sucrose, urea and glycerol at 25°C. Journal of the American Chemical Society 60: 3061-3070.

12. Gekko K, Timasheff SN (1981) Mechanism of Protein Stabilization by Glycerol - Preferential Hydration in Glycerol-Water Mixtures. Biochemistry 20: 4667-4676.

13. Schneider CP, Trout BL (2009) Investigation of Cosolute-Protein Preferential Interaction Coefficients: New Insight into the Mechanism by Which Arginine Inhibits Aggregation. Journal of Physical Chemistry B 113: 2050-2058.

14. Courtenay ES, Capp MW, Anderson CF, Record MT (2000) Vapor pressure osmometry studies of osmolyte-protein interactions: Implications for the action of osmoprotectants in vivo and for the interpretation of "osmotic stress" experiments in vitro. Biochemistry 39: 4455-4471.

15. Vagenende V, Yap MGS, Trout BL (2009) Mechanisms of Protein Stabilization and Prevention of Protein Aggregation by Glycerol. Biochemistry 48: 11084-11096.

16. Bhat TN, Bentley GA, Boulot G, Greene MI, Tello D, et al. (1994) Bound Water-Molecules and Conformational Stabilization Help Mediate an Antigen-Antibody Association. Proceedings of the National Academy of Sciences of the United States of America 91: 1089-1093.

17. MacKerell AD, Bashford D, Bellott M, Dunbrack RL, Evanseck JD, et al. (1998) All-atom empirical potential for molecular modeling and dynamics studies of proteins. Journal of Physical Chemistry B 102: 3586-3616.

18. Jorgensen WL, Chandrasekhar J, Madura JD, Impey RW, Klein ML (1983) Comparison of Simple Potential Functions for Simulating Liquid Water. Journal of Chemical Physics 79: 926-935.

19. Vagenende V, Yap MGS, Trout BL (2009) Molecular Anatomy of Preferential Interaction Coefficients by Elucidating Protein Solvation in Mixed Solvents: Methodology and Application for Lysozyme in Aqueous Glycerol. Journal of Physical Chemistry B 113: 11743-11753.

20. Baynes BM, Trout BL (2003) Proteins in mixed solvents: A molecular-level perspective. Journal of Physical Chemistry B 107: 14058-14067.

21. Reiling S, Schlenkrich M, Brickmann J (1996) Force field parameters for carbohydrates. Journal of Computational Chemistry 17: 450-468.

22. Phillips JC, Braun R, Wang W, Gumbart J, Tajkhorshid E, et al. (2005) Scalable molecular dynamics with NAMD. Journal of Computational Chemistry 26: 1781-1802.

23. Humphrey W, Dalke A, Schulten K (1996) VMD: Visual molecular dynamics. Journal of Molecular Graphics 14: 33-38.

1.  [↑](#footnote-ref-2)
